# Effects of Water Immersion and Soil Moisture Content on Larval and Pupal Survival of *Bactrocera minax* (Diptera: Tephritidae)

**DOI:** 10.3390/insects10050138

**Published:** 2019-05-14

**Authors:** Zaiyuan Li, Consolatha Chambi, Tianhua Du, Cong Huang, Fulian Wang, Guifen Zhang, Chuanren Li, Mohamed Juma Kayeke

**Affiliations:** 1 Forewarning and Management of Agricultural and Forestry Pests, Hubei Engineering Technology Center, College of Agriculture, Yangtze University, Jingzhou 434025, China; zaiyuanli01@163.com (Z.L.); dutianhua01@163.com (T.D.); huangcongchangda@163.com (C.H.); 13986706558@163.com (C.L.); 2 Department of Land-use Planning and Management, Ministry of Agriculture, Dodoma 2182, Tanzania; consolatha_chambi@yahoo.com; 3 State Key Laboratory for Biology of Plant Diseases and Insect Pests, Key Laboratory of Integrated Pest Management of Crop, Ministry of Agriculture and Rural Affairs, Institute of Plant Protection, Chinese Academy of Agricultural Sciences, Beijing 100193, China; 4 Ministry of Agriculture Tanzania Agricultural Research Institute, Mbeya 400, Tanzania; jkayeke@yahoo.com

**Keywords:** *Bactrocera minax*, soil water content, respiration rate, water immersion

## Abstract

*Bactrocera minax*, one of the most devastating citrus pests in Asia, has two developmental stages (mature larva and pupa) that complete their life cycle in the soil. Currently, southern China has a climate with abundant autumn rains, and soil moisture can be a major factor affecting the survival of larvae and pupae of *B. minax*. In the present study, we evaluated the effects of water immersion and high soil moisture content on the development of mature larvae and pupae of *B. minax*. When immersed in water for 1 d, 100% of mature larvae of *B. minax* were knocked out. When larvae were immersed for less than 6 d, however, more than 92% of knocked-out larvae recovered within 24 h. The days of water immersion with 50% and 90% recovery ratios (indicated as RD_50_ and RD_90_) were 10.3 d and 6.4 d, respectively. When larvae were immersed less than 6 d, the mortality ratios of larvae were not significantly different from those that were not immersed at all. The days of immersion causing 50% and 90% mortality of larvae (MD_50_ and MD_90_, respectively) were 7.6 d and 11.1 d, respectively. The pupation ratios of larvae were also observed to be not significantly different compared to non-immersion, and the days of immersion causing 50% and 90% pupation (PD_50_ and PD_90_, respectively) were 6.6 d and 0.8 d, respectively. Larval respiration rates were reduced after water immersion as a strategy for larval survival. High water content was not detrimental to pupae of *B. minax*. Adult emergence did not significantly decrease in soil with high water content, even though pupae were under those conditions for 161–175 d. The respiration rates of pupae were lower in soil with different moisture levels and were not significantly different, which ensured the survival of pupae in high water content. Reduced respiration rate is a strategy for survival of larvae and pupae, and remarkable tolerance to high moisture conditions could explain the high rate of spread and geographical distribution of *B. minax*. The results of this study provide a reference for the occurrence and control of *B. minax*.

## 1. Introduction

The respiratory organs of insects consist of tracheal tubes with external spiracular valves that control gas exchange [1]. Many insects have discontinuous patterns of gas exchange, and the respiratory system of insects consists of a highly branched system of cuticle-lined tubes extending throughout the body [2], which may have initially evolved in underground insects to deal with hypoxic conditions [3]. Soil oxygen concentrations are affected by moisture content [4]. Eskafi and Fernandez [5] identified the lack of oxygen as an important factor for increased mortality in pupa of *Ceratitis capitata*, when moisture content reached a saturation point. Hetz and Bradley [1] reported decreased respiration of underground insects.

Behavioral and physiological adaptations for surviving moist environments are important life traits for terrestrial invertebrates. Tolerance of insects to water immersion is different among species. For example, *Cieindela togata* has a maximum larval survival time of 6 d and time to 50% mortality of 85.9 h, which is more than eight times longer than those of similarly treated larvae of *Tenebrio molitor* immersed in water [6]. Mature larvae of *Anastrepha suspense* could be held in water for 12 h with no marked effect on emergence, and mortality reached 10% and 20% when larvae were immersed for 16 h and 24 h, respectively [7]. 

Soil moisture is one of the major lethal factors during the pupa period for several fruit fly species [8,9,10]. Pupae of *Bactrocera dorsalis* (Hendel) [11] and *B. correcta* (Bezzi) [12] cannot survive 100% soil moisture conditions, while the same moisture content causes only 30% pupal mortality in *B. tryoni* (Froggatt) [10]. Usually, pupal mortality increases with increasing moisture content in soil [13,14]. Pupation behavior of soil-pupating insects is also influenced by excessive soil water content. For example, when selecting a pupation site, mature larvae of the oriental fruit fly, *B. dorsalis*, prefer moist soil conditions [15]. The emergence ratio in *B. minax* was highest under 15–20% absolute soil moisture content [16]. 

*Bactrocera minax* (Enderlein), the Chinese citrus fly, is one of the most important pests of commercial citrus [17]. The hosts of *B. minax* include wild and cultivated citrus species, which are endemic in Southern China and the Eastern Himalayan region. This insect can also damage meiwa kumquat, *Fortunella crassifolia Swingle* cv. *chintan*, and trifoliate orange, *Poncirus trifoliate* [17,18]. *B. minax* has been reported in Southern China, India (West Bengal and Sikkim), and Bhutan [17,19]. *B. minax* is seriously harmful to the host plant, with maggot-infested fruits accounting for between 20% and 75% [18,20,21] and occasionally reaching 100% in some mountainous areas of China [17]. Chinese citrus fly populations are largely distributed in areas at 25 to 32 degrees north latitude [17] that have a subtropical monsoon climate with high rainfall [22].

*B. minax* has four developmental stages. The developmental phases of larva and pupa are the longest, and during these stages, *B. minax* are in contact with or live in the soil. Usually, maggot-infested fruits drop from the trees at the end of September–early November in orchards and the larvae live in the fruit until they are fully developed to the third instar, after which they burrow into the soil to pupate. The following spring, late April to early May, adults emerge from the soil [23].

Southern China has weather with abundant autumn rains [24], during which the soil moisture content is high, and the citrus orchards are often waterlogged, with 14.15–29.71% absolute water content [25,26]. In addition, citrus orchards are grown in mountainous areas where small rivers provide natural conditions for larval movement by the waterway systems, which is the primary pathway for the spread of *B. minax* [27,28]. Consequently, mature larvae are often immersed in water and the pupae are exposed to high moisture content soils because of abundant precipitation. The effect on survival of larval and pupal immersion in soil with different moisture contents have not been studied for *B. minax.*

In this study, we aimed to determine: (1) The effects of immersion of *B. minax* larvae on survival and pupation; (2) the effects of four different moisture levels of soils on emergence of *B. minax* pupae; and (3) the mechanisms for survival of larvae and pupae after immersion in wet soil through measuring the respiration rates of larvae and pupae. Such information may provide support for the distribution of *B. minax* in wet conditions and suggest a control option for *B. minax*.

## 2. Materials and Methods 

### 2.1. Insects 

Fallen citrus fruit infested with *B. minax* larvae from an orchard were collected and brought to the laboratory. A total of 4500 dropped grapefruits (*Citrus paradisi*) infested with *B. minax* larvae were brought to the Yangtze University insectary from an orchard (N 30°08.610′, E 111°62.191′) in Songzi, Hubei Province, China, in early November, 2015. Third-instar larvae (15–18 mm in length) were collected from the grapefruits. On 7 November 2015, a total of 3750 larvae (3rd instar) were used for the water immersion experiment. The rest of the larvae were allowed to pupate in soil (95.4% relative moisture content, equivalent to 15% absolute water content; soil type is sand with grain size of 0.3 mm). 

### 2.2. Effects of Water Immersion on B. minax Larvae 

Third-instar larvae were separated into fifteen cohorts. To assess the effects of water immersion on larvae, a total of 50 larvae were immersed in 50 mL distilled water in a glass beaker (200 mL, 7 cm in diameter) for 0–14 d. There were five replicates for each number of immersed days from 1 to 14; 50 larvae were used per replicate. After preparation, all beakers with larvae were placed indoors under natural conditions (temperature 10–17 °C, 64–95% relative humidity and 10:14 h light:darkness (L:D) photoperiod). After water immersion, the larvae were allowed to recover in plastic containers (15 cm × 10 cm × 8 cm) with soil. The soil type was sand with a grain size of about 0.3 mm and 15% absolute water content (the weight of sand was 1225.67 g and the weight of water was 183.85 g). All containers were placed indoors under natural conditions (temperature 10–17 °C, 64–95% relative humidity and 10:14 h (L:D) photoperiod).

The number of knocked-out larvae (no movement when touched with a brush) was recorded immediately after the water-immersion treatment. The numbers of recovered (active) individuals were counted after taking them out of the water 24 h later. The larvae were checked daily until all the larvae pupated or died, and the numbers of normal and dead pupae were recorded.

The amount of carbon dioxide (CO_2_) released by larvae was immediately measured using the LI-6400 infrared gas analyzer (IRGA)-chamber system (Li-Cor Inc., Lincoln, NE, USA) after different numbers of immersed days. This system precisely measured the CO_2_ in the incoming air flux and in the air leaving the sample chamber. Temperature (20 °C) and flow rate (100 μmols^−1^) within the sample chamber were controlled. There were three to five replicates for measuring the CO_2_ in each treatment; one larva was used per replicate. The computer associated with the IRGA system calculates assimilation or production of CO_2_ by the sample using the general gas exchange formula of Cooper and McLetchie [29].

### 2.3. Effect of Soil Moisture Content on the B. minax Pupae

We determined the effects on pupae immersed at an early stage and a late stage. When the pupae were immersed at an early stage, newly formed pupae were collected on 15 November 2015, and separated into five cohorts. To assess the effects of soil water content on pupae, a total of 30 newly formed pupae were transferred onto soil (soil type was sand, field capacity was measured at 15.72%) in a plastic container (dimensions: 15 cm × 10 cm × 8 cm). The soil had a grain size of about 0.3 mm, and five absolute water contents were used: 0% (water: 0 g, soil: 1225.67 g), 5% (water: 61.28 g, soil: 1225.67 g), 10% (water: 122.57 g, soil: 1225.67 g), 15% (water: 183.85 g, soil: 1225.67 g), and 20% (water: 245.13 g, soil: 1225.67 g). The soil water content was maintained by covering the plastic containers with a piece of perforated plastic film (pore diameter 0.5 mm, the number of pores was 36) and changed weekly. Pupae were buried in the soil at a depth of 5–10 cm. There were seven replicates of soil with different water contents; 30 newly formed pupae were provided per replicate. In early May 2016, the numbers of emerged adults were counted eight times daily (from 6:00 to 20:00) until no emergence was observed for 14 successive days.

When the pupae were immersed at a late stage, pupae (1st stage pupae, white colored adults initially formed inside and separated from the puparium [30]) were collected on 25 April 2016, and separated into five cohorts. A total of 100 1st stage pupae were transferred onto soil (soil type was sand) in a plastic container (dimensions: 15 cm × 10 cm × 8 cm). There were three replicates of soils with different water contents; 100 1st stage pupae were provided per replicate. The treatment of soil moisture content was the same as that in the early stage.

During both experiments, all containers were placed indoors under natural conditions (temperatures ranging from 5–23 °C, relative humidities of 64–100%, and natural sunlight conditions).

The amount of carbon dioxide (CO_2_) released by pupae was measured every 5 d using the LI-6400 infrared gas analyzer (IRGA) chamber system (Li-Cor Inc., Lincoln, NE, USA), when the pupae emerged at the early stage. There were three to five replicates for measuring the CO_2_ released by pupae in soil with different moisture contents, and one pupa was provided per replicate. Respiration rates of pupae in soils of different moisture contents were obtained by the average of CO_2_ released for the entire pupa stage.

### 2.4. Statistical Analysis

#### 2.4.1. Water Immersion on the Performance of Mature Larvae

After the larvae were immersed in water, they were knocked out (immobile larvae). The ratio of knocked-out larvae to total larvae was obtained by the following formula: 

“Knocked out ratio” = (Number of knocked-out larvae / Total number of immersed larvae) × 100. 

To assess the recovered ratio of knocked-out larvae in 24 h after removing them from the water, the following formula was used: 

“Recovered ratio” = (Number of recovered larvae in 24 h after removal from water/Total number of knocked-out larvae) × 100. 

The recovered ratio of knocked-out larvae was analyzed using logistic regression (Y_R_ = A/(1 + Exp(b − kX))) [31] where X was the number of immersed days and Y_R_ was the larvae recovered ratio in 24 h in probability units. The immersed days and recovered ratio data were fitted using probability analysis to estimate RD_50_ and RD_90_ (the number of immersed days that induced, respectively, 50% and 90% of knocked-out larvae to recover).

To assess the mortality of larvae caused by immersion, the following expression was used: 

“Larvae mortality ratio” = (Larvae mortality / Total number of tested larvae) × 100. 

The larvae mortality ratios were analyzed using logistic regression (Y_M_ = A/(1 + Exp(b − kX))) [31] where X was the immersed days and Y_M_ was the larvae mortality ratio in probability units. The immersed days and larval mortality data were fitted using probability analysis to estimate MD_50_ and MD_90_ (the number of immersed days that induced death in, respectively, 50% and 90% of larvae).

To assess the pupation ratio of larvae in different days of water immersion, the following expression was used:

“Pupation ratio” = (Number that pupated normally/Total number of tested larvae) × 100. 

The pupation ratios were analyzed using logistic regression (Y_P_ = A/(1 + Exp(b − kX))) [31] where X was the immersed days and Y_P_ was the pupation ratio in probability units. The immersed days and pupation ratio data were fitted using probability analysis to estimate PD_50_ and PD_90_ (the number of immersed days that induced, respectively, 50% and 90% of larvae to become pupae).

To assess the duration of pre-pupation after larvae were immersed, the following formula was used: 

“Pre-pupation duration” = (Σ pre-pupation days per pupae)/Total number pupated.

#### 2.4.2. Soil Moisture Content on Pupal Survival

To assess the effects of soil moisture on pupal survival, the following formula was used: 

“Adult emergence ratio” = (Number of adults emerged / Total number of pupae tested) × 100.

#### 2.4.3. Data Analysis

Percentage data were arcsine square-root transformed, and homogeneity of variance of all data was tested before one-way analysis of variance (ANOVA). All data were subjected to ANOVA, followed by Tukey’s multiple range test (*p* < 0.05) for significant differences between treatments. All statistical analysis was performed using SPSS 17.0.

## 3. Results

### 3.1. Effect of Water Immersion on Survival of Mature Larvae

**Figure 1:**
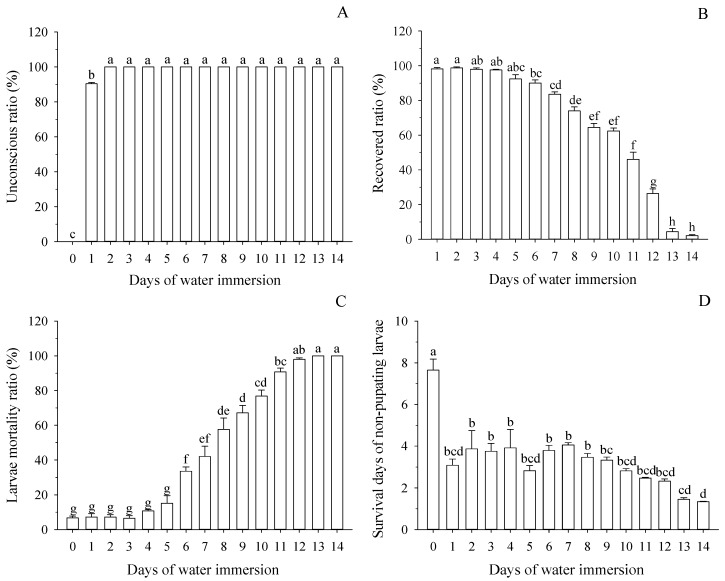
Effects of water immersion on the performance of mature larvae. (**A**): Ratio of knocked-out larvae in different days of water immersion. (**B**): Recovery ratio of knocked-out larvae in 24 h after removal from the water. (**C**): Mortality of larvae in different days of water immersion. (**D**): Survival days of non-pupated immersed larvae in different days of water immersion. Means (±SE) among different days of water immersion followed by different letters differ at *p* < 0.05 (Tukey HSD test).

After being immersed in water for 1 d, 90.4% immersed larvae were knocked out. When immersed for 2–14 d, the knocked out ratios were 100% when the larvae were taken out from water (Figure 1A). However, the number of days immersed had a significant effect on the recovery ratio (in 24 h after removal from the water) (*F* = 143.73; *df* = 13,55; *p* < 0.001; Figure 1B). When immersed for 1–5 d, more than 92% of knocked-out larvae recovered within 24 h. There was a significant decrease in the recovery ratio when larvae were immersed over 5 d. Recovery ratios were only 4.4% and 2.0% when the larvae were immersed for 13 and 14 d, respectively. The number of immersed days and the recovery ratio were fitted using logistic nonlinear regression: Y_R_=96.669/(1+Exp(−6.814+0.656X)) (*R* = 0.988; *R*_0.05_ = 0.532, *R*_0.01_ = 0.661) (Table 1 and Table 2). The days of water immersion with 50% and 90% recovery ratios (RD_50_ and RD_90_, respectively) were 10.3 d and 6.4 d, respectively (Table 3).

The days of water immersion had a significant effect on larval mortality (*F* = 143.73; *df* = 13,55; *p* < 0.001; Figure 1C). When larvae were immersed for 1–5 d, the mortality ratios of larvae (7.2–15.2%) were not significantly different from non-immersed larvae (6.8%; *p* > 0.967). However, the larval mortality began to increase significantly when larvae were immersed for 6 d, and the mortality ratio of larvae increased by 26.80% for 6 d of immersion compared to non-immersed larvae (6.8%; *p* < 0.001). When immersed over 12 d, the larval mortalities were 100%. The relationship between the days of water immersion and mortality ratio of larvae was determined by a logistic relationship and described by nonlinear regression: Y_M_ = 106.543/(1 + Exp(4.028 − 0.514X)) (*R* = 0.997; *R*_0.05_ = 0.514, *R*_0.01_ = 0.641) (Table 1 and Table 2). The days of immersion causing 50% and 90% mortality of larvae (MD_50_ and MD_90_, respectively) were 7.6 d and 11.1 d, respectively (Table 3).

The days of water immersion had a significant effect on the survival days of non-pupated larvae (*F* = 14.01; *df* = 14,58; *p* < 0.001; Figure 1D). After immersion, the survival days of non-pupated larvae decreased by 4.6 d (immersed for 1 d, *p* < 0.001) to 6.3 d (immersed for 14 d, *p* < 0.001) compared with non-immersed larvae.

### 3.2. Effect of Water Immersion on Pupation of Mature Larvae

**Figure 2:**
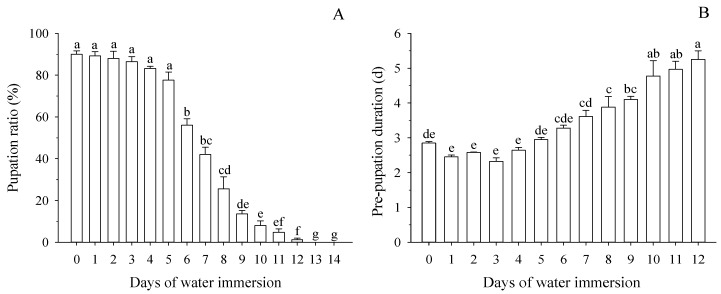
Effects of water immersion on the performance of pupation. (**A**): Pupation ratio of larvae in different days of water immersion. (**B**): Pre-pupation duration of larvae in different days of water immersion. Means (±SE) among different lengths of immersion followed by different letters differ at *p* < 0.05 (Tukey HSD test).

Pupation ratios differed in larvae with different days of water immersion (*F* = 147.48; *df* = 14,59; *p* < 0.001; Figure 2A). The pupation ratios of larvae immersed for 1–5 d were observed to be more than 77.60%, which was not significant compared to non-immersion (90.00%; *p* > 0.196). However, the pupation ratios began to decrease significantly when larvae were immersed for 6 d (*p* < 0.05), and the pupation ratios for the 6 d immersion decreased by 34.0% compared to the pupation ratios (90%) of non-immersed larvae (*p* < 0.001). There was no pupation after larvae were immersed in water for 13 or 14 d. The relationship between the days of water immersion and pupation ratio was determined by a logistic relationship and was described by nonlinear regression: Y_P_ = 90.735/(1 + Exp(−5.448 + 0.799X)) (*R* = 0.999; *R*_0.05_ = 0.532, *R*_0.01_ = 0.661) (Table 1 and Table 2). The days of immersion causing 50% and 90% pupation (PD_50_ and PD_90_, respectively) were 6.6 d and 0.8 d, respectively (Table 3).

There was a significant increase in the duration of pre-pupation with increasing days of water immersion (*F* = 27.950; *df* = 12,58; *p* < 0.001; Figure 2B). The duration of pre-pupation was not significant compared to non-immersion (*p* > 0.645), when the larvae were immersed for 1–7 d. However, the pre-pupation durations were significantly increased when larvae were immersed over 7 d compared to non-immersion (*p* < 0.005). 

### 3.3. Effect of Water Immersion on Respiration Rates of Mature Larvae

**Figure 3:**
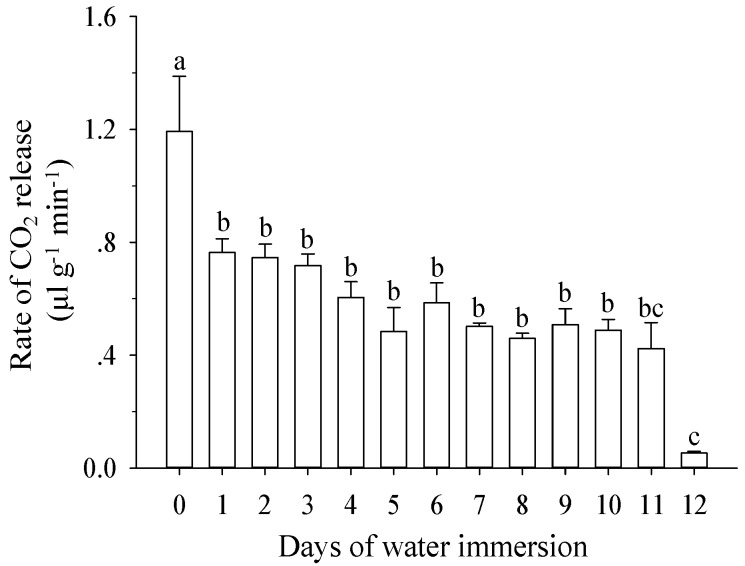
Effects of water immersion on larval respiration rates. Means (±one SE) among different days of water immersion followed by different letters differ at *p* < 0.05 (one-way ANOVA, Tukey test).

Once immersed in water, the respiration rates of larvae decreased significantly (*F* = 12.21; *df* = 12,49; *p* < 0.001; Figure 3). As the number of immersed days increased, the release of carbon dioxide (CO_2_) decreased by 0.43 μL/g/min (immersed for 1 d, *p* = 0.014) to 1.14 μL/g/min (immersed for 12 d, *p* < 0.001) compared to non-immersion (1.19 μL/g/min). However, there was no significant decrease in CO_2_ release when individuals were immersed for 2–11 d (*p* > 0.115).

### 3.4. Effects of Soil Moisture on Pupal Survival

**Figure 4:**
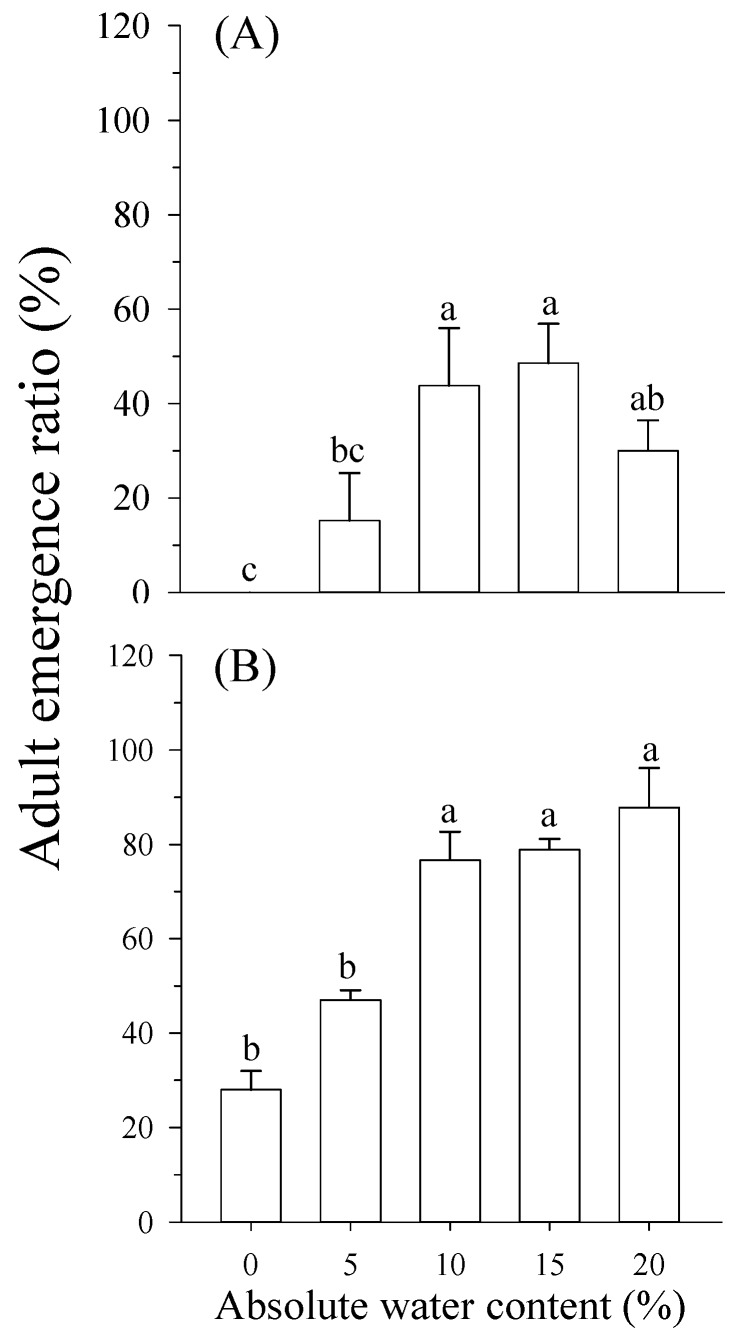
Adult emergence of pupae immersed in soil at different moisture levels. (**A**): Adult emergence from pupae immersed at the early stage. (**B**): Adult emergence from pupae immersed at the late stage. Means (±SE) among different soil moistures followed by different letters differ at *p* < 0.05 (Tukey HSD test).

Adult emergence was affected by soil moisture on early stage pupae (*F* = 9.885; *df* = 4,34; *p* < 0.000; Figure 4A). There was not adult emergence when pupae were immersed in 0%. The most suitable absolute water contents for adult emergence were 10% and 15%. Emergence was negatively affected when pupae were immersed in 5% (5% vs. 10%, *p* = 0.036; 5% vs. 15%, *p* = 0.018), but higher water content (20%) did not significantly decrease emergence (20% vs. 10%, *p* = 0.797; 20% vs. 15%, *p* = 0.621; Figure 4A), even though pupae were under those conditions for 161–175 d.

Adult emergence was affected by soil moisture on the late stage pupa (*F* = 15.300; *df* = 4,14; *p <* 0.000; Figure 4B). There was a higher emergence ratio when the soil absolute water content was 10% or 15%. Higher moisture (20%) was not detrimental to pupae of *B. minax* (20% vs. 10%, *p* = 0.583; 20% vs. 15%, *p* = 0.721; Figure 4B). However, adult emergence decreased greatly (5% vs. 10%, *p* = 0.028; 5% vs. 15%, *p* = 0.020), when the soil moisture was 5%. In particular, there was adult emergence when the pupae were immersed in 0%, which was the lowest (28%).

### 3.5. Effects of Soil Moisture on Respiration Rates of Pupae

**Figure 5:**
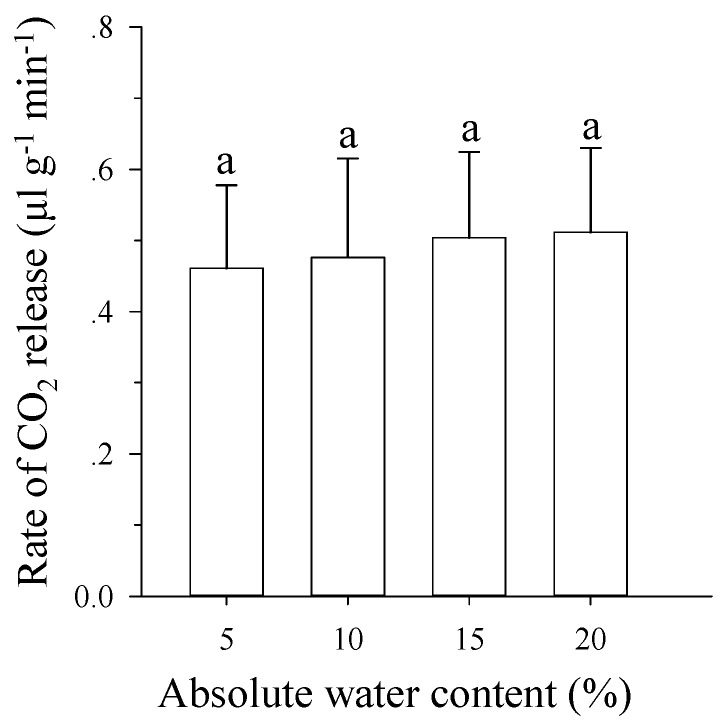
Effects of soil moisture on pupae respiration rates. Means (±SE) among different levels soil moisture followed by different letters differ at *p* < 0.05 (Tukey HSD test).

There were no significant differences in respiration rates for different soil moisture levels on pupae during the entire pupa stage (*F* = 0.036; *df* = 3,35; *p* = 0.991; Figure 5). The rates of CO_2_ release were 0.46, 0.47, 0.50, and 0.51 μL/g/min, respectively, for absolute water contents of 5%, 10%, 15%, and 20%.

## 4. Discussion

After leaving the host fruit, mature larvae of many fruit fly species wander around for time periods up to days (dispersal period) before burrowing into the soil [10,15]. Larvae of *B. minax* remain in the fallen fruit for 3−7 d (Fulian Wang and Guifen Zhang, personal observation). During this period, the larvae often live in an environment of excessive water because it is rainy in Southeast China during autumn [24]. In this study, after being immersed in water, 100% of larvae were knocked out (Figure 1A). However, when larvae were immersed for less than 6 d, more than 92% of knocked-out larvae recovered in 24 h after being removed from the water (Figure 1B). In addition, the larvae mortality ratios were no more than 15.2% when larvae were immersed for 1−5 d. The effect of maximum immersion time on insect survival varies based on insect species and development stage. Sun et al. [32] discovered that when *Carposina sasakii* pupae were immersed for 2 d, the mortality was 100%. Cavallaro et al. [33] found that the lethal time to 50% mortality for *Nicrophorus investigator*, *N. marginatus*, *Necrodes surinamensis*, and *Thanatophilus lapponicus* was 14.9, 9.0, 3.3, and 12.2 h, respectively. However, for *B. minax*, the numbers of immersed days causing 50% and 90% mortality of larvae (MD_50_ and MD_90_, respectively) were 7.6 d and 11.1 d, respectively (Table 3). Furthermore, the pupation ratios of larvae were found to be more than 77.60% within 1−5 d after immersion in water, which was not different compared to non-immersed larvae. These results indicate that the *B. minax* larvae have stronger tolerance to water immersion than some previously studied insects. There was a slight shortening in the pre-pupation period, when the larvae were immersed to water for 1–4 d (Figure 2B). This phenomenon indicates that short-term immersion can shorten the development duration of larvae. Liu et al. [34] discovered that short-term immersion promoted egg development in the grasshopper *Fruhstorferiola tonkinensis*. In summary, these results indicate that surviving larvae of *B.* *minax* have remarkable tolerance to water immersion.

In this study, the larvae respiration rates decreased when larvae were immersed in water (Figure 3). We suggest that the lower respiration rates are one survival strategy for *B. minax* larvae forced into water immersion. However, larvae would die if they were immersed in water over the maximum saturation time, because the amount of oxygen for *B. minax* respiration could be insufficient. Similarly, Eskafi and Fernandez [5] identified lack of oxygen as an important factor of drastically increased pupae mortality in *Ceratitis capitata* when moisture content reached a saturation point. 

The insect survival is not only associated with immersion period, but also insect density and amount of water in water immersion environment [35,36]. We investigated the effect of immersion period on the survival of citrus large fruit fly larvae, and the larval density and water content need to be further studied

The emergence of *B. minax* adults from soils with different moisture levels showed that pupae survival was not affected by high water content. Even in pupae exposed to high moisture (20%) throughout the entire pupa stage, adult emergence was not significantly affected. This meant that *B. minax* pupae also had a remarkably high tolerance to high moisture stress.

When *B. minax* spent the entire pupa stage in soil with 15% or 20% absolute water content (above 95.4% relative water content), emergence was 48.6% and 30.0%, respectively. These were lower than that of *B*. *tryoni* (68−78%) [10], but higher than that of *Anastrepha ludens* (Loew) or *A. obliqua* (Macquart) (4−10%) [37]. No adults of *B*. *correcta* emerged in 100% relative water content conditions [12] and *B. dorsalis* pupae did not survive in such conditions [11]. The differences in species and/or soil types may account for such differences. 

In the present study, soil water content did not affect the respiration rates of *B. minax* pupae (Figure 5). By contrast, the respiration rates of pupae were lower than larva for *B. minax* (*p* < 0.022). There was a period of diapause during the entire pupa stage [38], and Wang et al. [38] found that the respiration rates of pupae were low during the period of diapause compared to larvae for *B. minax*. In hypoxic environments, insects may rely on anaerobic metabolism and metabolic depression to survive [33,39]. Underground environments are potentially hypoxic. Hetz and Bradley [1] found that insects often reduce respiration to adapt to hypoxic underground environments. So, we suggest that *B. minax* pupae survived in the high water content soil by maintaining lower respiration rates, which decreased with the oxygen requirement for the entire pupa stage.

However, we found that lower soil moisture content had more detrimental effects on adult emergence of *B. minax* compared to high moisture content. Similarly, the emergence ratio of *A*. *ludens* was lower (3.2%) when the pupae were immersed in dry soil [37]. Hulthen and Clarke [10] found that 85% pupal mortality of *B*. *tryoni* occurs in dry soil (0% field capacity). The emergence ratio of *B. dorsalis* in dry soil (0% water content) increased by 55.5−88.9% compared to soil with 100% relative water content [14], which indicated that *B. dorsalis* had remarkable tolerance to dry soil. Thus, dry soil (lower moisture content) may be more harmful than soil with high moisture content to *B. minax* populations in the field. We suggest that the high mortality of pupae in dry soil is due to the fact that the development of pupae without adequate water supply when the pupae are immersed in soil with lower moisture content. This is consistent with the observations of Hou et al. [40] who found that in drought conditions, soil moisture content was too low to meet the requirements for pupal development of *B. dorsalis*, and larvae went deeper into the soil to seek water. Furthermore, insect respiration is one of the causes of water loss and gain in the body, so pupae deaths are attributed to the fact that pupae lose too much water and cannot obtain enough water from outside the body when the pupae are immersed in soil of low moisture content.

Our results showed that *B. minax* pupae have higher emergence ratios when immersed in soil with different moisture levels (representing drought to excessive water conditions) on the late stage pupa than those of pupae immersed throughout the entire pupa stage. These results agree in part with those obtained by Ni et al. [41] who found that when *B*. *correcta* pupae were immersed in water, survival was affected by the pupae stage, and there was little influence of water immersion on older pupae compared to younger pupae, which showed that older pupae had stronger tolerance to high water content. Wang et al. [38] found that the metabolic rates of pupae at the late stage were higher and also determined that the respiration rates of pupae at the late stage were higher than pupae immersed for the entire pupa stage. So, we suggest that the demand for water is higher for pupae at the late stage, which is one of the reasons for a high death rate for pupa immersed for the entire pupa stage. In addition, there is a special structure for pupae at the late stage: white color adults initially formed inside and separated from the puparium [30], which enhances the resistance to low water content for pupae at the late stage. 

In an integrated pest management program, even though the larvae and pupae have remarkable tolerance, the larvae mortality ratio significantly increased with increased days of immersion, and low moisture content soil may be more harmful to *B. minax* populations than soil with high moisture content. Collecting maggot-infested fruits has proven to be an effective control measure against *B. minax* because it reduces the number of pupae in the field. *B. minax*-infested fruits can be treated by immersing them in water for a long time to control larvae. This measure may also help to slow down the spread of this fruit fly species to uninfected areas.

## 5. Conclusions

South China has a subtropical monsoon climate, which is dominated by rains in October; during this time most *B. minax*-infested fruits drop from the trees in orchards. This study indicates that larvae of *B. minax* seemed remarkably tolerant to immersion in water and thus, larvae inside the dropped fruit immersed in rain or irrigation water for a few days would not be negatively affected. *B. minax* is thought to be primarily spread by the waterway systems [27,28], and the stronger tolerance of larvae immersed in water provide support for that. This increased the possibility of further spread of *B. minax* if the infested fruits are carried by rivers or mountain streams. In addition, the present study also indicated that pupae had a stronger tolerance to soil of high moisture content, and this ability is a prerequisite for the survival of the pests in areas with a subtropical monsoon climate. This provides reasons for the distribution of *B. minax* in areas with wet subtropical monsoon climates. This ability may also increase the invasive potential of this species, especially in areas with a wet winter. Low respiration may account for the tolerance of immersion or higher moisture soil and the survival of *B. minax* in subtropical monsoon climate areas. 

## Figures and Tables

**Table 1:** Curve equation from a regression between immersed days and recovery ratio of knocked-out larvae within 24 h, larvae mortality ratio, and pupation ratio of larvae of *B. minax*.

Parameter	Regression Equation	*R*	*R*_0.05_	*R*_0.01_
Recovery ratio	Y_R_ = 96.669/(1 + Exp(−6.814 + 0.656X))	0.988	0.532	0.661
Larvae mortality ratio	Y_M_ = 106.543/(1 + Exp(4.028 − 0.514X))	0.997	0.514	0.641
Pupation ratio	Y_P_ = 90.735/(1 + Exp(−5.448 + 0.799X))	0.999	0.532	0.661

*Y_R_ is the recovery ratio of knocked-out larvae in 24 h after removal from the water, Y_M_ is the mortality of larvae caused by immersion, and Y_P_ is the pupation ratio of larvae in different days of water immersion. X is the immersed days, *R* is the coefficient of determination; *R*_0.05_ is the coefficient of determination at 95%, *R*_0.01_ is the coefficient of determination at 99%. (*R* greater than *R*_0.05_ and *R*_0.01_ shows a strong relationship between immersed days and recovered ratio, larvae mortality ratio, and pupation ratio).

**Table 2:** Estimated parameters, confidence intervals, and *R*^2^ for a logistic model of recovery ratio, larvae mortality ratio, and pupation ratio fitted to experimental data of immersed *B. minax* larvae.

Parameter	Estimated	95% CI	*R* ^2^
Recovery ratio			
A	96.669	(90.616, 102.722)	0.977
b	−6.814	(−8.860, −4.768)	
k	−0.656	(−0.842, −0.470)	
Larvae mortality ratio			
A	106.543	(99.429, 113.657)	0.994
b	4.028	(3.503, 4.552)	
k	0.514	(0.432, 0.595)	
Pupation ratio			
A	90.735	(88.184, 93.286)	0.998
b	−5.448	(−6.115, −4.780)	
k	−0.799	(−0.888, −0.710)	

Note: A, b, and k represent the parameters in the logistic equation (Y = A/(1 + Exp(b − kX))).

**Table 3:** Time estimates for 50 and 90% of recovery ratio, larvae mortality ratio, and pupation ratio.

Parameter	Time Estimates			
	RD_50_/MD_50_/PD_50_		RD_90_/MD_90_/PD_90_	
	Days	(95% CI)	Days	(95% CI)
Recovery ratio	10.3	(−5.9, 26.5)	6.4	(−24.6, 37.4)
Larvae mortality ratio	7.6	(−1.6, 16.8)	11.1	(−6.5, 28.7)
Pupation ratio	6.6	(−0.6, 13.7)	0.8	(−12.7, 14.3)

Note: RD_50_ is the immersed days that induced 50% of knocked-out larvae to recover, MD_50_ is the immersed days that caused death to 50% of larvae, PD_50_ is the immersed days that induced 50% of larvae to become pupae, RD_90_ is the immersed days that induced recovery in 90% of knocked-out larvae, MD_90_ is the immersed days that caused death to 90% of knocked-out larvae, and PD_90_ is the immersed days that induced 90% of knocked-out larvae to become pupae. CI is the confidence interval.
